# The Role of Imaging Biomarkers Derived From Advanced Imaging and Radiomics in the Management of Brain Tumors

**DOI:** 10.3389/fonc.2020.559946

**Published:** 2020-09-23

**Authors:** Faiq Shaikh, Diana Dupont-Roettger, Jamshid Dehmeshki, Omer Awan, Olga Kubassova, Sotirios Bisdas

**Affiliations:** ^1^Image Analysis Group, Philadelphia, PA, United States; ^2^Department of Computer Science, Kingston University, Kingston-upon-Thames, United Kingdom; ^3^Department of Radiology, University of Maryland Medical Center, Baltimore, MD, United States; ^4^Department of Neuroradiology, University College London, London, United Kingdom

**Keywords:** imaging, biomarkers, radiomics, brain, tumors

## Introduction

Significant advances have been made in the realm of medical image analysis in the past few decades, aimed at improving our understanding of the disease—how it develops, behaves, and responds to treatment. Advanced imaging strategies using magnetic resonance imaging (MRI) and positron emission tomography (PET) provide structural and functional phenotypic biomarkers that correlate with key disease processes. Radiomics-based biomarkers provide a deeper analysis of pathophysiologic processes and insights to better diagnose, classify, stratify, and prognosticate brain tumors, and to assess their response to therapy.

### Radiomics in Neuro-Oncology

Radiomics is an imaging analysis methodology that involves the extraction of quantifiable features, which serve as biomarkers for structural changes as well as pathophysiological processes in disease entities. Applying radiomics yields a numerical dataset that can be parsed, processed, and analyzed using machine learning methods (1). Radiomics-based biomarkers can provide key insights in the diagnosis, classification, and therapeutic management of various solid tumors. It is also beginning to have an impact in the management of neuro-oncological diseases, including low-grade gliomas, glioblastoma multiforme (GBM), and brain metastases (2). There is a wide spectrum of radiomics applications in this field, ranging from accurate classification of brain lesions (gliomas vs metastases, IDH-wild type vs. -mutant tumors), therapy planning (radiation therapy response prediction), and immunotherapy response assessment.

### Methodology

Radiomics analysis may be performed on computed tomography (CT), magnetic resonance imaging (MRI), positron emission tomography (PET), and single-photon emission computed tomography (SPECT). Lesion identification and image segmentation are performed as the first steps and can be a manual or automated process, followed by 3D reconstruction performed on these regions of interest.

**Figure 1:**
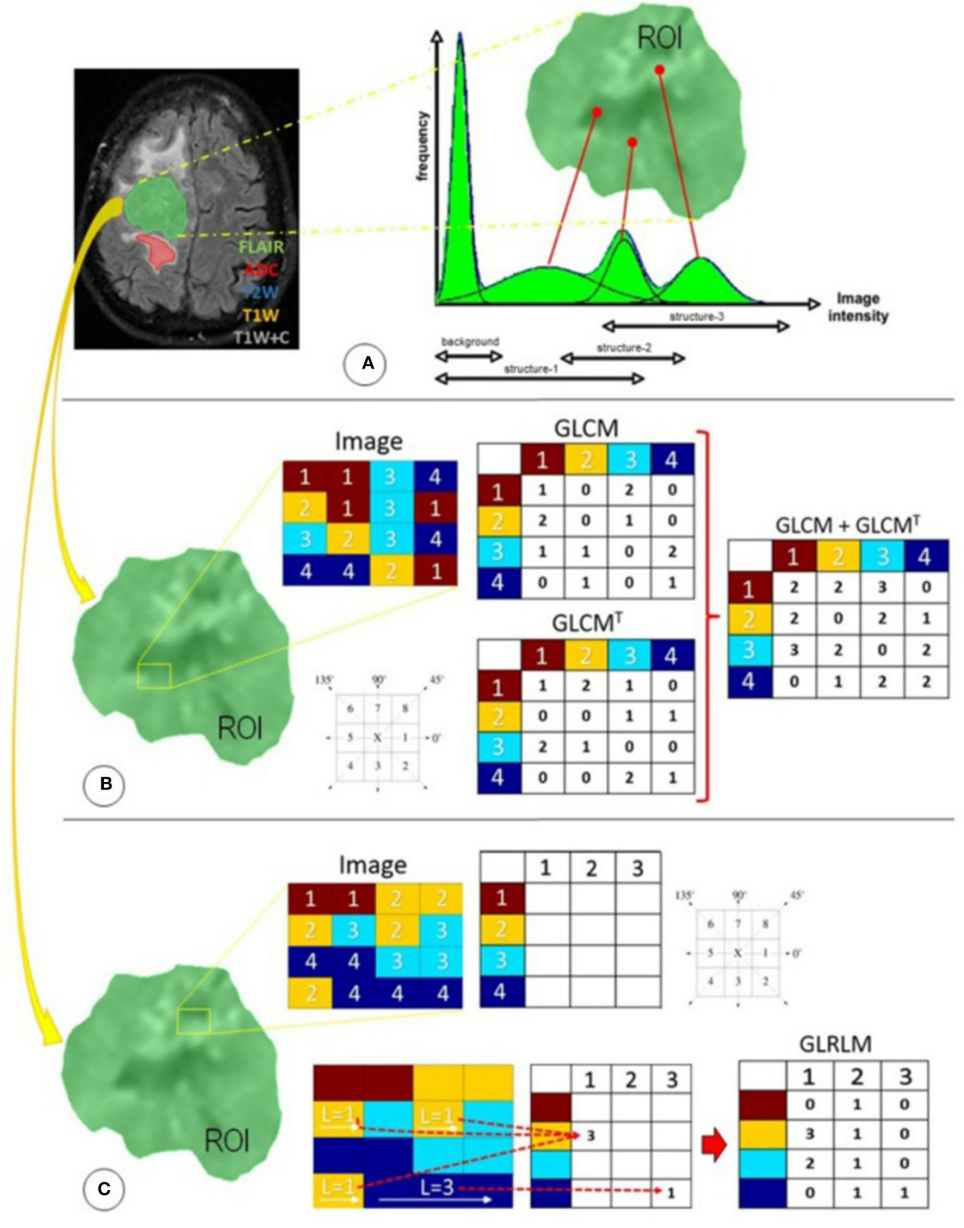
Radiomics features used in this study were distributed in three different techniques focused primarily on statistical approaches: **(A)** first-order statistics, **(B)** second-order statistics through the GLCM, and **(C)** higher-order statistics through the GLRLM. ADC, apparent diffusion coefficient; FLAIR, fluid-attenuated inversion recovery; GLCM, gray-level co-occurrence matrix; GLCMT, gray-level co-occurrence matrix transpose; GLRLM, gray-level run-length matrix; L, length of homogeneous runs for each gray level; ROI, region of interest; T1W, T1-weighted precontrast; T1W+C, T1-weighted postcontrast; T2W, T2-weighted. (Reused from Florez E, Nichols T, E Parker E, T Lirette S, Howard CM, Fatemi A. Multiparametric magnetic resonance imaging in the assessment of primary brain tumors through radiomic features: a metric for guided radiation treatment planning. *Cureus*. (2018) 10:e3426. doi: 10.7759/cureus.3426, under the CC-BY license).

The next step is that of feature extraction and classification (FE/FC). These features are categorized as shape features (morphology-based), first-order statistics (histogram-based), and second-order statistics (texture analysis) features (3). Furthermore, higher-order statistics may also be extracted using mathematical transforms (such as Minkowski functionals, Laplace features, wavelet transforms, etc.) (4). Feature extraction produces several numerical values (depending on the imaging modality and the library used for extraction), which are then analyzed using advanced statistical or machine learning (ML) approaches, which may be supervised or unsupervised, and include cluster analysis, support vector machine (SVM), random forest, convolutional neural network (CNN), and deep learning neural network (DLNN) (see Figure 1) (5). The main purpose is to train a model to identify radiomics features that can serve as imaging biomarkers for disease processes. This is followed by model validation and includes methods such as *k*-fold cross-validation to test the skill of the ML model. More recent works in neuro-oncology involving DLNN have revolved around automated tumor segmentation, quantification of disease burden, pseudoprogression assessment, multi-omics-based disease characterization, and prognostication.

## Discussion

### The Current Imaging Biomarker Landscape in Neuro-Oncology

A noninvasive imaging biomarker may be described as a characteristic feature identifiable on an imaging study that indicates a key disease process. The key step is to establish these new biomarkers through correlation with ground truths, which could be the previously imaging-based “gold standards,” clinical outcomes, or pathologic evidence. There is an increasing fund of quantitative imaging biomarkers (QIB) that are catalyzing the practice of precision medicine (6). In clinical trials, the QIBs are being used as surrogate endpoints, which can significantly reduce the time and incurred costs (7). QIBs are being explored as predictive classifiers for clinical trials, which can be used for patient selection/recruitment and in the timely determination of responders vs. nonresponders.

Brain lesions are structurally and functionally complex, and there is a growing focus on noninvasive methods to study this complexity to assess the disease status. Gliomas are a heterogeneous set of tumors, based on their issue, cellular, and molecular characteristics. The role of nonimaging biomarkers in gliomas and GBMs is well known, i.e., IDH1 mutation (8) and methylguanine-DNA methyltransferase (MGMT) promoter methylation (9). However, the role of imaging biomarkers in disease stratification or management guidance of GBM is less established.

Multiple imaging biomarkers have been identified for brain metastasis from various primary tumors. Multiparametric MRI, which includes apparent diffusion coefficient (ADC) and perfusion-weighted sequences, is used extensively in the clinical management of brain tumors. Perfusion-weighted and permeability MRI have been used for detection, delineation, and therapy response assessment of malignant brain lesions (10). Dynamic susceptibility contrast-enhanced MRI (DSC-MRI) deriving relative cerebral blood volume (rCBV) and cerebral blood flow (rCBF) values have led the quantifiable image biomarker discovery (11). Higher rCBV in the peritumoral edema, which may contain infiltrating angiogenic tumor cells, is indicative of primary intrinsic tumor as opposed to pure vasogenic edema seen in metastatic disease (12). However, the evidence for ADC to do the same is weak. Also, rCBV measurement from the solid tumoral region is another established discriminative biomarker for distinguishing GBM from the other tumor types (13).

Magnetic resonance spectroscopy (MRS) allows us to assess tissue metabolites noninvasively and has yielded several biomarkers of interest, such as choline (Cho)/creatinine (Cr) ratio, which is, for example, lower in cerebral metastases than in GBMs (14). Similarly, the peritumoral Cho/NAA ratio has also been shown to be useful to that effect (15). Furthermore, decreased creatine/phosphocreatine (Cr) values in patients with low-grade gliomas (WHO grade II) have been shown to correlate with better prognosis in terms of longer progression-free times and later malignant transformation (16). High levels of glycine have been reported in biopsies of patients with GBM (17). These, among other metabolites such as lactate, have been implicated as important MRS-based biomarkers for brain tumors.

18F-Fluorodeoxyglucose (FDG) PET/CT and more recently PET/MR has traditionally had a limited role in the management of primary brain tumors, primarily due to FDG biodistribution in the brain, hence there is an increasing role for amino-acid PET tracers in neuro-oncology. 18F-Fluoro-ethyl-tyrosine (FET) has been shown to detect recurrence in previously treated glioblastomas and is influenced by MGMT promoter methylation status (18). FET-PET-based biological tumor volume in newly diagnosed GBM has been shown to be a prognostic imaging biomarker for survival, independent of MGMT promoter methylation (19). However, it is important to note that the role of this biomarker for survival outcomes modeling has not been established. High tumoral amino-acid uptake using 11C-methionine (MET) PET is another well-studied biomarker for malignant gliomas and is independently associated with poor prognosis (20). α-[^11^C]Methyl-L-tryptophan PET has also been shown to predict longer overall survival (21).

### Radiomics-Based Imaging Biomarkers in Neuro-Oncology: A Novel Paradigm

Radiomic signatures are providing the next-generation imaging biomarkers that have implications in the management of brain tumors. These signatures are based on combinations of first-order histogram-based features (Haralik features, kurtosis, and entropy) and second-order texture analysis features (such as gray-scale run lengths). Key areas in neuro-oncology where radiomics has been initially applied are the following:

- ***Precision diagnostics and disease stratification/classification:***Since primary and metastatic brain tumors are histologically and genetically heterogeneous, and it is important to understand the role tumor heterogeneity plays in the natural history of cancer, its response to therapy, and prognosis/outcomes (22–24). The extracted radiomics features provide a numerical value for the heterogeneous tumor microenvironment changes (25). GBM is a notoriously aggressive cancer, given its therapeutic resistance and high recurrence rate, both of which have underpinnings in its molecular heterogeneity (26). Radiomics has provided insights into the tissue and molecular heterogeneity and correlated with the underlying genetic alterations (27–29). Furthermore, molecular heterogeneity of GBM at the transcriptomic level can be assessed using radiomics and may provide a framework to classify/stratify GBMs (30). Shofty et al. (31) demonstrated the ability of radiomics analysis of multiparametric MRI to stage 1p/19q co-deleted low-grade gliomas with sensitivity, specificity, and accuracy of 92, 83, and 87%, respectively (31). There is an interest in reclassifying many cancer types from the conventional histological basis to that based on radiogenomic signatures shedding light into various tissue heterogeneity patterns as they are better aligned with therapeutic responsiveness (32, 33).
- ***Disease prognostication and prediction modeling:***There are multiple prognostic determinants for brain tumors, including the histologic subtype, specific genetic mutations, degree of anaplasia, degree of necrosis of fibrosis, degree of de-differentiation, local infiltration, vasculogenesis and resulting vascular scavenging, and hypoxia. For most of these processes, radiomics analysis can provide some degree of quantification, such as wavelet transforms for the degree of vascularity or Minkowski functionals for the degree of necrosis (34). Zhang et al. (35) demonstrated the use of Minkowski features among others to help differentiate radiation necrosis from tumor progression in patients with brain metastases undergoing gamma-knife surgery. MR-based radiomics analysis has been shown to predict overall survival and progression-free survival in GBM (36). Radiomics signatures correlate with and predict the expression of key molecular biomarkers in brain tumors, such as Ki-67 expression in low-grade gliomas or IDH mutation in GBM (37, 38). These early predictive models may provide bases of re-classifying cancers based on their progression and prognosis, allowing indolent cancers to be managed more conservatively while reserving more aggressive therapeutic approaches for more aggressive cancers. This is exemplified by a study by Davatzikos et al. where they showed that molecular features depicted by radiomics provided better risk stratification of GBM beyond the WHO classification (39). Furthermore, radiomics can help in the assessment of medical complications associated with brain tumors, such as epilepsy in patients with low-grade gliomas, which facilitates better disease management (40).
- ***Therapy response assessment and monitoring:***Radiomics-based phenotype assessment of cancer lesions is an effective tool in the sensitivity profiling against therapeutic options (such as quantifying hypoxia to determine chemosensitivity), as well as an early assessment of therapy response (41–43). The standard visual assessment of radiological images for this purpose has been plagued by the confounding pseudoprogression. Current MRI techniques and human-based interpretation are tedious and prone to high interpersonal variability for accurate classification and prognostication of gliomas (44). The current Response Assessment in Neuro-oncology (RANO) criteria are used for GBM and the immunotherapy RANO (iRANO) criteria have been introduced to address the issue of pseudoresponse/pseudoprogression for both conventional chemoradiation and immunotherapies (45). Novel approaches using multiparametric MR and/or PET imaging combined with radiomics-based texture analysis can help evaluate subtle microstructural as well as functional changes at earlier time points than standard imaging (46). These can be quantifiable harbingers of true therapy response assessment and debunking pseudo-progression more accurately and earlier than conventional approaches. A multicenter study performed by Elshafeey et al. (47) using MR-based radiomics analysis for immunotherapy response assessment in GBM yielded an accuracy, specificity, and sensitivity of 91, 91, and 88%, respectively.

Radiomic tumor signatures can be incorporated into a multidimensional, multi- “omics” model, which uses genetic/molecular determinants to create a holistic genotype–phenotype the landscape of cancer and have the potential for informing the prognosis and accurately predicting/assessing therapy response (48). New approaches, such as using Multi Assay Experiment (MAE) as the container for multi-omics analysis, facilitates the process of data compilation and integration required for such complex analyses (49). Furthermore, quantitative scoring scales based on such ML-based analytical models have applications in clinical management as well as “go/no-go” decision-making in clinical trials (50, 51).

Generally speaking, there are factors that hinder the full-scale application and widespread acceptance of radiomics in the field of neuro-oncology. This includes the lack of user-friendly, FDA-approved software programs that perform radiomic analysis, the lack of a generalizable model to use for predictions, and the lack of a prospective study to show the added value of radiomics compared with the conventional ROI and histogram analysis.

Specific to its clinical application in neuro-oncologic management, there are certain gaps where radiomics has yet to make an impact. These include providing biomarkers for the precision guidance of the therapeutic management of brain tumors, particularly GBM. Genetic markers that are implicated in the prognostication and therapy guidance of GBM include gene amplification of epidermal growth factor receptor (EGFR), TP53, and PTEN mutation, among others (52, 53). Developing radiomic signatures that correlate with these genetic markers can help develop noninvasive imaging biomarkers for risk/severity stratification, survival outcomes, and therapy response prediction and assessment for these patients. Furthermore, having such radiomic biomarkers can catalyze the development of novel therapeutics using these genetic markers as targets.

The scope of radiomics applications is growing. When vetted through robust statistical analyses and real-world applications, it can augment the shift toward personalized, precision-based practices in neuro-oncology.

## Author Contributions

FS: conceptualization, subject expertise (cancer imaging + radiomics methodology). SB: subject matter expertise (clinical neuroradiology). OK: subject matter expertise (mathematics, statistics). OA: subject matter expertise (clinical imaging). JD: subject matter expertise (computer science, machine learning methodology). DD-R: subject matter expertise (computer vision methodology). All authors contributed to the article and approved the submitted version.

## Conflict of Interest

FS, DD-R, OK, JD, and SB are full-time/part-time employees of Image Analysis Group. The remaining author declares that the research was conducted in the absence of any commercial or financial relationships that could be construed as a potential conflict of interest.

## References

[1] Gillies RJ Kinahan PE Hricak H. Radiomics: images are more than pictures, they are data. Radiology. (2016) 278:563–77. 10.1148/radiol.2015151169 26579733 PMC4734157

[2] Zhou M Scott J Chaudhury B Hall L Goldgof D Yeom KW. Radiomics in brain tumor: image assessment, quantitative feature descriptors, and machine-learning approaches. Am J Neuroradiol. (2018) 39:208–16. 10.3174/ajnr.A5391 28982791 PMC5812810

[3] Parmar C Velazquez ER Leijenaar R Jermoumi M Carvalho S Mak RH. Robust radiomics feature quantification using semiautomatic volumetric segmentation. PLoS One. (2014) 9:e0102107. 10.1371/journal.pone.0102107 25025374 PMC4098900

[4] Kumar V Gu Y Basu S Berglund A Eschrich SA Schabath MB. Radiomics: the process and the challenges. Magn Reson imaging. (2012) 30:1234–48. 10.1016/j.mri.2012.06.010 22898692 PMC3563280

[5] Parmar C Grossmann P Bussink J Lambin P Aerts HJ Machine learning methods for quantitative radiomic biomarkers. Sci Rep. (2015) 5:13087 10.3389/fonc.2015.00272 26278466 PMC4538374

[6] Larue RT Defraene G De Ruysscher D Lambin P Van Elmpt W. Quantitative radiomics studies for tissue characterization: a review of technology and methodological procedures. Br J Radiol. (2017) 90:20160665. 10.1259/bjr.20160665 27936886 PMC5685111

[7] O'Connor JP Jackson A Asselin MC Buckley DL Parker GJ Jayson GC. Quantitative imaging biomarkers in the clinical development of targeted therapeutics: current and future perspectives. Lancet Oncol. (2008) 9:766–76. 10.1016/S1470-2045(08)70196-7 18672212

[8] Turkalp Z Karamchandani J Das S. IDH mutation in glioma: new insights and promises for the future. JAMA Neurol. (2014) 71:1319–25. 10.1001/jamaneurol.2014.1205 25155243

[9] Weller M Stupp R Reifenberger G Brandes AA Van Den Bent MJ Wick W. MGMT promoter methylation in malignant gliomas: ready for personalized medicine?. Nat Rev Neurol. (2010) 6:39. 10.1038/nrneurol.2009.197 19997073

[10] Lacerda S Law M. Magnetic resonance perfusion and permeability imaging in brain tumors. Neuroimaging Clin. (2009) 19:527–57. 10.1016/j.nic.2009.08.007 19959004

[11] Paulson ES Schmainda KM. Comparison of dynamic susceptibility-weighted contrast-enhanced MR methods: recommendations for measuring relative cerebral blood volume in brain tumors. Radiology. (2008) 249:601–13. 10.1148/radiol.2492071659 18780827 PMC2657863

[12] Chiang IC Kuo YT Lu CY Yeung KW Lin WC Sheu FO. Distinction between high-grade gliomas and solitary metastases using peritumoral 3-T magnetic resonance spectroscopy, diffusion, and perfusion imagings. Neuroradiology. (2004) 46:619–27. 10.1007/s00234-004-1246-7 15243726

[13] Xing Z You RX Li J Liu Y Cao DR. Differentiation of primary central nervous system lymphomas from high-grade gliomas by rCBV and percentage of signal intensity recovery derived from dynamic susceptibility-weighted contrast-enhanced perfusion MR imaging. Clin Neuroradiol. (2014) 24:329–36. 10.1007/s00062-013-0255-5 23994941

[14] Tsougos I Svolos P Kousi E Fountas K Theodorou K Fezoulidis I. Differentiation of glioblastoma multiforme from metastatic brain tumor using proton magnetic resonance spectroscopy, diffusion and perfusion metrics at 3 T. Cancer Imaging. (2012) 12:423. 10.1102/1470-7330.2012.0038 23108208 PMC3494384

[15] Server A Josefsen R Kulle B Mæhlen J Schellhorn T Gadmar Ø. Proton magnetic resonance spectroscopy in the distinction of high-grade cerebral gliomas from single metastatic brain tumors. Acta Radiol. (2010) 51:316–25. 10.3109/02841850903482901 20092374

[16] Hattingen E Raab P Franz K Lanfermann H Setzer M Gerlach R. Prognostic value of choline and creatine in WHO grade II gliomas. Neuroradiology. (2008) 50:759–67. 10.1007/s00234-008-0409-3 18523762

[17] Choi C Ganji SK DeBerardinis RJ Dimitrov IE Pascual JM Bachoo R. Measurement of glycine in the human brain *in vivo* by 1H-MRS at 3 T: application in brain tumors. Magn Reson Med. (2011) 66:609–18. 10.1002/mrm.22857 21394775 PMC3139742

[18] Munck af Rosenschold P Costa J Engelholm SA Lundemann MJ Law I Ohlhues L. Impact of [18F]-fluoro-ethyl-tyrosine PET imaging on target definition for radiation therapy of high-grade glioma. Neuro Oncol. (2015) 17:757–63. 10.1093/neuonc/nou316 25537018 PMC4482858

[19] Suchorska B Jansen NL Linn J Kretzschmar H Janssen H Eigenbrod S. Biological tumor volume in 18FET-PET before radiochemotherapy correlates with survival in GBM. Neurology. (2015) 84:710–9. 10.1212/WNL.0000000000001262 25609769

[20] De Witte O Goldberg I Wikler D Rorive S Damhaut P Monclus M. Positron emission tomography with injection of methionine as a prognostic factor in glioma. J Neurosurg. (2001) 95:746–50. 10.3171/jns.2001.95.5.0746 11702862

[21] John F Bosnyák E Robinette NL Amit-Yousif AJ Barger GR Shah KD. Multimodal imaging-defined subregions in newly diagnosed glioblastoma: impact on overall survival. Neuro Oncol. (2019) 21:264–73. 10.1093/neuonc/noy169 30346623 PMC6374760

[22] Fidler IJ. Tumor heterogeneity and the biology of cancer invasion and metastasis. Cancer Res. (1978) 38:2651–60. 354778

[23] Charles NA Holland EC Gilbertson R Glass R Kettenmann H. The brain tumor microenvironment. Glia. (2011) 59:1169–80. 10.1002/glia.21136 21446047

[24] Sottoriva A Spiteri I Piccirillo SG Touloumis A Collins VP Marioni JC. Intratumor heterogeneity in human glioblastoma reflects cancer evolutionary dynamics. Proc Natl Acad Sci U S A. (2013) 110:4009–14. 10.1073/pnas.1219747110 23412337 PMC3593922

[25] Lambin P Rios-Velazquez E Leijenaar R Carvalho S Van Stiphout RG Granton P. Radiomics: extracting more information from medical images using advanced feature analysis. Eur J Cancer. (2012) 48:441–6. 10.1016/j.ejca.2011.11.036 22257792 PMC4533986

[26] Parker NR Khong P Parkinson JF Howell VM Wheeler HR. Molecular heterogeneity in glioblastoma: potential clinical implications. Front Oncol. (2015) 5:55. 10.3389/fonc.2015.00055 25785247 PMC4347445

[27] Cui Y Tha KK Terasaka S Yamaguchi S Wang J Kudo K. Prognostic imaging biomarkers in glioblastoma: development and independent validation on the basis of multiregion and quantitative analysis of MR images. Radiology. (2016) 278:546–53. 10.1148/radiol.2015150358 26348233 PMC4734164

[28] Yang D Rao G Martinez J Veeraraghavan A Rao A. Evaluation of tumor-derived MRI-texture features for discrimination of molecular subtypes and prediction of 12-month survival status in glioblastoma. Med Phys. (2015) 42:6725–35. 10.1118/1.4934373 26520762 PMC5148162

[29] Sala E Mema E Himoto Y Veeraraghavan H Brenton JD Snyder A. Unravelling tumour heterogeneity using next-generation imaging: radiomics, radiogenomics, and habitat imaging. Clin Radiol. (2017) 72:3–10. 10.1016/j.crad.2016.09.013 27742105 PMC5503113

[30] Kong DS Kim J Ryu G You H-J Sung JK Han YH. Quantitative radiomic profiling of glioblastoma represents transcriptomic expression. Oncotarget. (2018) 9:6336–45. 10.18632/oncotarget.23975 29464076 PMC5814216

[31] Shofty B Artzi M Bashat DB Liberman G Haim O Kashanian A. MRI radiomics analysis of molecular alterations in low-grade gliomas. Int J Comput Assist Radiol Surg. (2018) 13:563–71. 10.1007/s11548-017-1691-5 29270916

[32] Louis DN Perry A Reifenberger G Von Deimling A Figarella-Branger D Cavenee WK. The 2016 World Health Organization classification of tumors of the central nervous system: a summary. Acta Neuropathol. (2016) 131:803–20. 10.1007/s00401-016-1545-1 27157931

[33] Colen RR Hassan I Elshafeey N Zinn PO. Shedding light on the 2016 World Health Organization Classification of Tumors of the Central Nervous System in the era of radiomics and radiogenomics. Magn Reson Imaging Clin. (2016) 24:741–9. 10.1016/j.mric.2016.07.001 27742114

[34] Sanduleanu S Woodruff HC De Jong EE Van Timmeren JE Jochems A Dubois L. Tracking tumor biology with radiomics: a systematic review utilizing a radiomics quality score. Radiother Oncol. (2018) 127:349–60. 10.1016/j.radonc.2018.03.033 29779918

[35] Zhang Z Yang J Ho A Jiang W Logan J Wang X A predictive model for distinguishing radiation necrosis from tumour progression after gamma knife radiosurgery based on radiomic features from MR images. Eur Radiol. (2018) 28:2255–63. 10.1007/s00330-017-5154-8 29178031 PMC6036915

[36] Chaddad A Sabri S Niazi T Abdulkarim B. Prediction of survival with multi-scale radiomic analysis in glioblastoma patients. Med Biol Eng Comput. (2018) 56:2287–300. 10.1007/s11517-018-1858-4 29915951

[37] Li Y Qian Z Xu K Wang K Fan X Li S. Radiomic features predict Ki-67 expression level survival in lower grade gliomas. J Neurooncol. (2017) 135:317–24. 10.1007/s11060-017-2576-8 28900812

[38] Li ZC Bai H Sun Q Zhao Y Lv Y Zhou J. Multiregional radiomics profiling from multiparametric MRI: Identifying an imaging predictor of IDH1 mutation status in glioblastoma. Cancer Med. (2018). 7:5999–6009. 10.1002/cam4.1863 30426720 PMC6308047

[39] Rathore S Akbari H Rozycki M Abdullah KG Nasrallah MP Binder ZA. Radiomic MRI signature reveals three distinct subtypes of glioblastoma with different clinical and molecular characteristics, offering prognostic value beyond IDH1. Sci Rep. (2018) 8:1–2. 10.1038/s41598-018-22739-2 29572492 PMC5865162

[40] Liu Z Wang Y Liu X Du Y Tang Z Wang K. Radiomics analysis allows for precise prediction of epilepsy in patients with low-grade gliomas. NeuroImage Clin. (2018) 19:271–8. 10.1016/j.nicl.2018.04.024 30035021 PMC6051495

[41] Aerts HJ Velazquez ER Leijenaar RT Parmar C Grossmann P Carvalho S. Decoding tumour phenotype by noninvasive imaging using a quantitative radiomics approach. Nat Commun. (2014) 5:1–9. 10.1038/ncomms5644 24892406 PMC4059926

[42] Vallières M Kay-Rivest E Perrin LJ Liem X Furstoss C Aerts HJ. Radiomics strategies for risk assessment of tumour failure in head-and-neck cancer. Sci Rep. (2017) 7:1–4. 10.1038/s41598-017-10371-5 28860628 PMC5579274

[43] Aerts HJ. The potential of radiomic-based phenotyping in precision medicine: a review. JAMA Oncol. (2016) 2:1636–42. 10.1001/jamaoncol.2016.2631 27541161

[44] Brandsma D Stalpers L Taal W Sminia P van den Bent MJ. Clinical features, mechanisms, and management of pseudoprogression in malignant gliomas. Lancet Oncol. (2008) 9:453–61. 10.1016/S1470-2045(08)70125-6 18452856

[45] Okada H Weller M Huang R Finocchiaro G Gilbert MR Wick W. Immunotherapy response assessment in neuro-oncology: a report of the RANO working group. Lancet Oncol. (2015) 16:e534–42. 10.1016/S1470-2045(15)00088-1 26545842 PMC4638131

[46] Da Cruz LH Rodriguez I Domingues RC Gasparetto EL Sorensen AG. Pseudoprogression and pseudoresponse: imaging challenges in the assessment of posttreatment glioma. Am J Neuroradiol. (2011) 32:1978–85. 10.3174/ajnr.A2397 21393407 PMC7964401

[47] Elshafeey N Kotrotsou A Hassan A Elshafei N Hassan I Ahmed S. Multicenter study demonstrates radiomic features derived from magnetic resonance perfusion images identify pseudoprogression in glioblastoma. Nat Commun. (2019) 10:1–9. 10.1038/s41467-019-11007-0 31320621 PMC6639324

[48] Bera K Beig N Tiwari P Opportunities and advances in radiomics and radiogenomics in neuro-oncology. In: *International Workshop on Radiomics and Radiogenomics in Neuro-oncology* Cham: Springer (2019), p. 12–23.

[49] Zanfardino M Franzese M Pane K Cavaliere C Monti S Esposito G. Bringing radiomics into a multi-omics framework for a comprehensive genotype-phenotype characterization of oncological diseases. J Transl Med. (2019) 17:337. 10.1186/s12967-019-2073-2 31590671 PMC6778975

[50] Pinker K Shitano F Sala E Do RK Young RJ Wibmer AG. Background, current role, and potential applications of radiogenomics. J Magn Reson Imaging. (2018) 47:604–20. 10.1002/jmri.25870 29095543 PMC5916793

[51] Bi WL Hosny A Schabath MB Giger ML Birkbak NJ Mehrtash A. Artificial intelligence in cancer imaging: clinical challenges and applications. CA Cancer J Clin. (2019) 69:127–57. 10.3322/caac.21552 30720861 PMC6403009

[52] Hill C Hunter SB Brat DJ. Genetic markers in glioblastoma: prognostic significance and future therapeutic implications. Adv Anat Pathol. (2003) 10:212–7. 10.1097/00125480-200307000-00004 12826827

[53] Schmidt MC Antweiler S Urban N Mueller W Kuklik A Meyer-Puttlitz B. Impact of genotype and morphology on the prognosis of glioblastoma. J Neuropathol Exp Neurol. (2002). 61:321–8. 10.1093/jnen/61.4.321 11939587

